# The Effect of Increased Intra-Abdominal Pressure on Hemodynamics in Laparoscopic Cholecystectomy—The Experience of a Single Centre

**DOI:** 10.3390/jpm14080871

**Published:** 2024-08-17

**Authors:** Elena Stamate, Alin-Ionut Piraianu, Oana-Monica Duca, Oana Roxana Ciobotaru, Ana Fulga, Iuliu Fulga, Cristian Onisor, Madalina Nicoleta Matei, Alexandru-Stefan Luchian, Adrian George Dumitrascu, Octavian Catalin Ciobotaru

**Affiliations:** 1 Department of Morphological and Functional Sciences, Faculty of Medicine and Pharmacy, “Dunarea de Jos” University of Galati, 35, Al. I. Cuza Street, 800216 Galati, Romania; elena.stamate@ugal.ro (E.S.); cristian.onisor@ugal.ro (C.O.); 2 Department of Clinical Medical, Faculty of Medicine and Pharmacy, “Dunarea de Jos” University of Galati, 35, Al. I. Cuza Street, 800216 Galati, Romania; roxana.ciobotaru@ugal.ro; 3 Department of Clinical Surgical, Faculty of Medicine and Pharmacy, “Dunarea de Jos” University of Galati, 35, Al. I. Cuza Street, 800216 Galati, Romania; ana.fulga@ugal.ro (A.F.); octavian.ciobotaru@ugal.ro (O.C.C.); 4 Department of Medical, Faculty of Medicine and Pharmacy, “Dunarea de Jos” University of Galati, 35, Al. I. Cuza Street, 800216 Galati, Romania; iuliu.fulga@ugal.ro; 5 Department of Dental Medicine, Faculty of Medicine and Pharmacy, “Dunarea de Jos” University of Galati, 35, Al. I. Cuza Street, 800216 Galati, Romania; madalina.matei@ugal.ro; 6 Emergency County Hospital Braila, 810325 Braila, Romania; luchian_alexandrustefan@yahoo.com; 7 Division of Hospital Internal Medicine, Department of Medicine, Mayo Clinic Florida, 4500 San Pablo Rd S, Jacksonville, FL 32224, USA; dumitrascu.adrian@mayo.edu

**Keywords:** minimally invasive surgery, cholecystectomy, intra-abdominal pressure, blood pressure, heart rate, cardiac output

## Abstract

Laparoscopic cholecystectomy is characterized by reduced postoperative pain, shorter hospital stays, rapid return to preoperative physical activity, and less psychological impact on the patient. During laparoscopic cholecystectomy, the intra-abdominal insufflation of carbon dioxide with secondary increase in intra-abdominal pressure can cause important hemodynamic consequences, like decreased cardiac output and blood pressure, as well as compensatory increase in heart rate. The purpose of this study is to evaluate changes in cardiovascular parameters during general anesthesia in patients undergoing laparoscopic cholecystectomy. Retrospective data from 342 patients with cholecystectomy for cholelithiasis performed at Railway Hospital Galati, Romania, were reviewed. All patients received the same intraoperative anesthetics. Female patients were 85.7% (*n* = 293). More than half of the patients, 53.51% (*n* = 183), were 40–59 years old, and only 16.37% (*n* = 56) were under 40 years old. Patients with a normal body mass index (BMI) represented 45.6% (*n* = 156), 33.3% (*n* = 114) were underweight, and 12% (*n* = 42) had grade 1 obesity (BMI 25–29.9 kg/m^2^). The minimum intraoperative blood pressure correlated with patient gender (*p* 0.015 < 0.005), with men having a higher blood pressure than women (*p* 0.006 < 0.05), and for BMI, a higher BMI was associated with elevated blood pressure (*p* 0.025 < 0.05). Older age correlated with an increased maximum intraoperative blood pressure (*p* < 0.001 < 0.05) and with maximum intraoperative heart rate (*p* 0.015 < 0.05). Patients undergoing laparoscopic cholecystectomy experienced significant hemodynamic changes with pneumoperitoneum, but this type of surgical intervention was safe for patients regardless of their age.

## 1. Introduction

Modern laparoscopy was introduced in the 1980s by Kurt Semm [1]. In 1882, Carl Langebuch of Germany performed the first cholecystectomy [2]. In 1985, 103 years later, Prof Dr Erich Mühe of Germany performed the first laparoscopic cholecystectomy [3]. Since then, minimally invasive surgery has become the gold standard in many diseases including symptomatic cholelithiasis [4], and it has become the approach of choice for most common surgical procedures due to its advantages, such as shorter hospital stays and faster recovery times [5]. Laparoscopic surgery has been widely used in recent decades because of its favorable short-term outcomes, such as reduced blood loss, improved recovery time, and less pain.

During laparoscopic surgery, the combined effect of body position, pneumoperitoneum, and anesthesia can alter patient’s respiratory and cardiovascular physiology [6].

The intra-abdominal insufflation of carbon dioxide in laparoscopic cholecystectomy can increase intra-abdominal pressure [6]. Initially, hemodynamic consequences due to increased intra-abdominal pressure include increased cardiac afterload and preload. Subsequently, as intra-abdominal pressure rises, it leads to the compression of the inferior vena cava, decreased venous return to the heart, and decreased cardiac output, potentially being followed by decreased perfusion to the coronary arteries and acute coronary syndrome [7,8].

In addition to hemodynamic changes during general anesthesia in laparoscopic cholecystectomy like decreased cardiac output, decreased blood pressure, and compensatory increase in heart rate, respiratory changes are also described in the literature. Ventilatory consequences include decreased pulmonary compliance, increased airway pressures, and hypercarbia [9]. Limited atelectasis occurs in almost all patients (90%) during general anesthesia [10]. This is the result of a reduction in functional residual capacity and a decrease in chest wall muscle tone [11]. In patients with an increased body mass, this anesthesia side effect is more pronounced due to lung compression by the intra-abdominal organs and the weight of the chest wall. The effects of pressure on the lungs, the reduction in functional residual capacity, and the reduction in lung compliance in laparoscopic abdominal procedures are consequences of pneumoperitoneum formation [12,13,14]. Thus, the four major pulmonary complications that can occur with abdominal carbon dioxide (CO_2_) insufflation for pneumoperitoneum include hypoxemia, hypercarbia, subcutaneous emphysema, and reduction in pulmonary compliance. Hypercarbia can cause myocardial depression, arrhythmias, the exacerbation of pulmonary hypertension, and systemic vasodilation [9].

Observing these hemodynamic changes in our post cholecystectomy patient population, we designed a study to evaluate changes in cardiovascular parameters during general anesthesia in patients undergoing laparoscopic cholecystectomy.

## 2. Materials and Methods

### 2.1. Data Collection and Subjects

We conducted a retrospective observational clinical study. After the application of selection criteria (≥18 years old, surgery for cholelithiasis diagnosis), 784 patients were found to be eligible. We excluded patients (*n* = 442) that were treated with open cholecystectomy. For patients with acute calculous cholecystitis, surgery was delayed until the inflammatory process subsided to allow for a safe laparoscopic intervention. Patients that had choledocholithiasis on ultrasound examination first underwent an endoscopic retrograde cholangiopancreatography (ERCP) to extract the stones from the bile ducts, with the laparoscopic cholecystectomy being performed subsequently [15]. Finally, a cohort of 342 eligible adult patients was included. All patients were admitted to the Railway Hospital in Galati, Romania, in the General Surgery Department, between January 2011 and March 2016, with the diagnosis of symptomatic cholelithiasis and underwent laparoscopic cholecystectomy. Our patients were from a single medical center, and general anesthesia was administered by the same doctor that also performed the postoperative, post-anesthesia follow-up.

Inclusion criteria: patients with symptomatic cholelithiasis undergoing laparoscopic cholecystectomy and receiving general anesthesia with intraoperative fentanyl analgesia.

Exclusion criteria: patients undergoing classic/open cholecystectomy; patients with acute cholecystitis, cholelithiasis, and choledocholithiasis; patients with any type of pulmonary pathology; patients with any type of renal pathology; patients with acute/chronic inflammatory diseases or thyroid pathology; and patients with any underlying chronic cardiological diagnosis other than hypertension.

In order to maintain intraoperative hemodynamic stability for patients with hypertension, renin–angiotensin–aldosterone system inhibitors and diuretics were stopped 12 h before surgery [16]. None of the patients included in our study took alpha-2 receptor agonist medication.

The patients were divided into three groups depending on age: <40 years; 40–60 years, and >60 years.

We examined the influence of demographic and clinical factors (gender, age, smoking history, body mass index—BMI) on cardiovascular parameters variation during laparoscopic cholecystectomy and in the immediate postoperative period. We classified patients according to BMI: normal weight (BMI = 18.5–24.9 kg/m^2^), overweight (BMI = 25–29.9 kg/m^2^), obesity class I (BMI = 30–34.9 kg/m^2^), obesity class II (BMI = 35–39.9 kg/m^2^), or obesity class III (BMI > 40 kg/m^2^) [17].

For operative risk prediction, we used the American Society of Anesthesiologists (ASA) Classification, taking in account patient’s associated pathology: normal healthy patient (ASA 1), mild systemic disease (ASA 2), severe systemic disease that is not life-threatening (ASA 3), severe systemic disease that is life-threatening (ASA 4), moribund patients with operation as the only solution for survival (ASA 5), and patients with irreversible brain damage whose organs are being removed for donor purposes (ASA 6) [18].

We identified the perioperative cardiovascular changes and determined their importance for patients undergoing general anesthesia and with operative pneumoperitoneum. For this, we monitored the following parameters: maximum and minimum heart rate and maximum and minimum blood pressure. Blood pressure monitoring was performed non-invasively: two measurements were taken preoperatively at 15 min, every 5 min intraoperatively, and postoperatively at 10 min in the first hour, and then at 30 min. All patients were followed for 24 h postoperatively.

### 2.2. Procedures

All patients had 6–8 h of liquid and food fasting prior to surgery. To initiate induction under normovolemic conditions, 10 mL/kg crystalloid infusion was administered preoperatively.

After preoxygenation, Propofol 2 mg/kg, fentanyl 3 μg/kg, and succinylcholine (1 mg/kg) were administered for induction, and anesthesia maintenance was achieved with atracurium 0.1 mg/kg at 20 min, fentanyl 2 μg/kg, repeated at 20–30 min, and sevoflurane. At awakening, pentazocine, atropine, and neostigmine were used.

To create the working chamber in the first stage of surgery, carbon dioxide was insufflated into the peritoneal cavity using a Veress needle at a pressure of 12 mmHg, pressure that was maintained during the surgery. This pressure could be reduced to 8–10 mmHg in case of hemodynamic deterioration.

For a better visualization of the gallbladder, patients were placed in reverse Trendelenburg supine position, with the torso elevated at 30 degrees and slightly tilted to the left.

During anesthesia, volume control ventilation was used. The patient was ventilated with a positive end-expiratory pressure (PEEP) of 5–7 cmH_2_O, a fraction of inspired oxygen (FiO_2_) = 0.5, a respiratory rate of 12 breaths/min, and a tidal volume (VT) of 8 mL/kg, to maintain an end-tidal of carbon dioxide (ETCO_2_) of approximately 38–40 mmHg and an oxygen saturation (SaO_2_) > 96 percent without exceeding peak pressures over 44 mmHg.

### 2.3. Statistical Analysis

The statistical processing of data was carried out using IBM (International Business Machines, Armonk, NY, USA) SPSS (Statistical Package for Social Sciences) software version 21. Both descriptive and analytical statistical methods were used for data processing.

The database consisted of numerical type data (BP, HR, age, weight, height, BMI), ordinal type data (age groups; blood pressure levels: <100 mmHg; 100–120 mmHg; 121–140 mmHg; >140 mmHg), and nominal type data (gender, smoker or non-smoker, rural or urban residence, elective surgery or emergent surgery, hypertensive diagnosis, antihypertensive medication use).

Descriptive analysis was performed using frequency tables, distributions, and crosstabs, for which mean values ± standard deviation and percentages were calculated.

Analytical statistics were performed, depending on the type of variables analyzed, using correlation coefficients.

For numerical variables, we calculated Pearson’s correlation coefficient, Kendall’s τb, γ (Gamma), and Somer’s d correlation coefficient.

For nominal variable correlation analysis, we calculated the nominal level correlation coefficients φ, C, and V, as well as conducted the χ^2^ test.

Comparisons between two or more independent groups were analyzed using the Mann–Whitney and Kruskal–Wallis tests.

We considered a significance threshold α = 0.05. The association between 2 or more variables was considered statistically significant when the obtained *p*-value was *p* < 0.05.

### 2.4. Results

**Figure 1:**
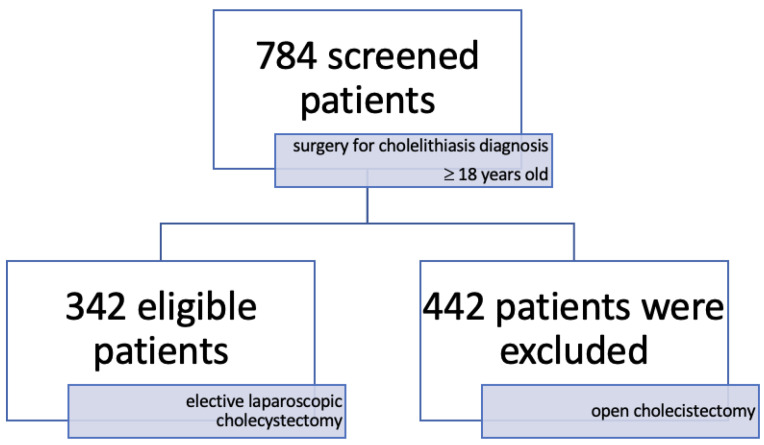
Flow chart of patient selection.

After the application of selection criteria and screening, 784 patients were screened, and (Figure 1) 342 patients that underwent laparoscopic cholecystectomy for cholelithiasis were enrolled. Among the 342 patients, 85.7% (*n* = 293) were female and 14.3% (*n* = 49) were male. More than half of the patients, 53.51% (*n* = 183), were in the 40–59 age group, and patients in the under 40 age group were the fewest with a share of only 16.37% (*n* = 56). Patients with a normal BMI represented 45.6% (*n* = 156), 33.3% (*n* = 114) were underweight, and 12% (*n* = 42) had grade 1 obesity (BMI 25–29.9). The most frequently encountered anesthetic risk was the American Society of Anesthesiologists physical status ASA 2 at 79.24% (*n* = 271).

Female patients accounted for 85.7% (*n* = 293), and male patients, for only 14.3% (*n* = 49).

When reviewing demographic factors, we found that patients living in urban areas 66.6% (*n* = 228) outnumbered those from rural areas 33.3% (*n* = 114).

Regarding the age distribution, half of the patients included in the study, namely 53.87% (*n* = 183), were in the age group of 40–59 years, followed by those in the age group of over 60 years, representing 30.12% (*n* = 103), with only 16.37% (*n* = 56) being under 40 years.

Most patients had a normal BMI, 43.7% (*n* = 156), closely followed by overweight patients, 33.57% (*n* = 114), and then the ones with grade 1 obesity with a percentage of 12.3% (*n* = 42), obesity grade II with a percentage of 2.34% (*n* = 8), and the lowest percentage being that of obesity grade 3 with a percentage of 3.2% (*n* = 1).

Patients that were actively smoking at the time of intervention accounted for 17.84% (*n* = 61), while non-smokers accounted for 82.2% (*n* = 281).

According to anesthetic risk, the majority of patients met ASA II 79.2% (*n* = 271), at a significant distance from those in ASA III 17% (*n* = 58), ASA I 3.5% (*n* = 12), and ASA IV 0.3% (*n* = 1).

Of all patients, 40.1% (*n* = 137) had a diagnosis of hypertension on presentation.

The data from the descriptive statistics are summarized in Table 1.

All patients had a favorable outcome, without any need for positive inotropic support, vasoconstrictors, or direct vasodilators either intraoperatively or postoperatively.

Analytical statistics highlighted the correlations between maximum and/or minimum blood pressure values, and heart rate with age, sex, BMI, and smoking status, respectively. The analysis was performed in the preoperative, intraoperative, and postoperative periods (Table 2).

## 3. Discussion

The major advantages of laparoscopic surgery over open surgery are less tissue damage, less pain induced by surgical wound closure, and shorter postinterventional bowel function recovery. These lead to better postoperative outcomes and lung function, a rapid recovery, and a shorter hospital stay. Cholecystectomies have become less invasive and safer over the past few decades even for pregnant women [19,20].

However, we have to take into account the physiological consequences induced by the increased intra-abdominal pressure, the absorption of the gasses used for insufflation, as well as the Trendelenburg position that is required during laparoscopic cholecystectomy.

In our study, women comprised the majority of patients (85.7%). This result was expected, knowing the increased incidence of cholelithiasis in women [21].

Also, we observed that 66.6% of patients were from urban living areas. Ye Rim Chang et al. conducted a study based on data obtained over 30 years from patients with gallstone disease in a single center in Korea and observed that, similar to our study findings, 67% of patients were living in urban areas [22].

Most patients (53.7%) were in the 40–59-year-old age group, and only 30.12% were over 60 years of age This finding is in contradiction with data from the literature that suggest that the prevalence of gallstones increases with age, being found in 50% of patients over 70 years old and only in 8% of those over 40 years old [22]. The explanation could pertain to our study criteria that excluded acute cholecystitis, an acute complication that develops more frequently in the elderly. 

According to the anesthetic risk, the majority (79.2%) of patients who underwent laparoscopic cholecystectomy were ASA II, indicating that most patients in our study were healthy or had only mild systemic disease. Our results are consistent with those of Tang Y.’s study, which showed that the majority of patients who underwent laparoscopic surgery (general abdominal, gynecological, or urological surgery) fell into the ASA I-II category [23].

In our study, we demonstrated a correlation between blood pressure and BMI, both in the preoperative and intraoperative period, a higher BMI being associated with a higher blood pressure. The mechanisms causing elevated blood pressure in obese patients are multifaceted, including the stimulation of the renin–angiotensin–aldosterone system, the overactivation of the sympathetic nervous system, insulin resistance, and alterations in adipose-derived cytokines [24]. During laparoscopic cholecystectomy, these mechanisms are amplified.

Mechanical and neurohormonal responses occur secondary to pneumoperitoneum. Hemodynamic changes during general anesthesia in laparoscopic cholecystectomy can occur due to increased abdominal pressure from pneumoperitoneum. Subsequently the increased intra-abdominal pressure causes the compression of the inferior vena cava, splenic and renal vasculature, and the aorta. Compression on the inferior vena cava leads to decreased venous return to the heart and consequently decreased cardiac beat volume [9]. Since cardiac output is the product of beat volume and heart rate, to maintain optimal cardiac output with decreasing beat volume, a compensatory increase in heart rate will occur.

The intraperitoneal pressure has a biphasic effect on the inferior vena cava pressure. Initially, beginning with an intraperitoneal pre-systolic pressure of 7.5 mmHg, this pressure causes an increase in inferior vena cava blood return pressure. This initial increase in pressure in the inferior vena cava is due to the “autotransfusion effect”/mobilization of the accumulated blood from the splanchnic circulation, leading to an increase in circulating blood volume, with an increase in pressure in the right atrium, then in the left atrium, and thus an increase in cardiac output [9].

A further elevation in intraperitoneal pressure above 15 mmHg decreases venous return to the inferior vena cava with lower limb stasis. The compression on the renal vessels with reduction in renal blood flow releases vasopressin, adrenaline, noradrenaline, and atrial natriuretic peptide. These hormones increase peripheral vascular resilience and blood pressure, thus increasing left ventricular afterload [25]. In our study, there were two situations where conversion to open surgery was necessary due to a significant reduction in blood pressure (BP < 100 mmHg) and a significant increase in ventilation pressure (Max inspiratory pressure > 20 mmHg).

Laparoscopy in patients with obesity may be associated with an increased risk of intra- and perioperative complications starting with intubation difficulties to mechanical ventilation with increased pressures and, more importantly, hemodynamic changes.

The respiratory function of patients with obesity is affected by increased oxygen consumption secondary to increased metabolism. They also have a predisposition for hypercapnia due to reduced thoracic wall compliance, reduced lung compliance, low reserve respiratory volumes, and decreased functional residual capacity [26]. Supine decubitus position and anesthesia add a reduction in functional residual capacity. Sometimes, a controlled ventilation with higher volumes and pressure maintenance parameters is needed to maintain oxygen and carbon dioxide blood partial pressure values within normal limits. Unfortunately, sometimes these values cannot be maintained. Studies show that in patients with morbid obesity, the vital capacity is reduced by 20%, and the expiratory reserve volume is reduced by 30 to 60% [27].

Increased intra-abdominal pressure secondary to CO_2_ insufflation causes diaphragm ascension with reduced functional residual capacity, with ventilator-empire mismatch that may result in hypoxia, which induces sympathetic stimulation and an increase in blood pressure. At the same time, the absorption of insufflated CO_2_ causes an increase in PCO_2_ with worsening of hypoxia by increasing this mismatch [28,29].

For the best visualization of the gallbladder, the patient is positioned in reverse Trendelenburg position, supine, and with the head elevated at a 30-degree angle, as well as with the body slightly rotated to the left. The reverse Trendelenburg position contributes to respiratory and cardiovascular changes. Respiratory mechanisms are improved by lowering the diaphragm and decreasing the resistance with increased lung compliance. From a circulatory point of view, it negatively affects hemodynamics by decreasing venous return and lower limb stasis [30].

A patient with obesity will have a higher rate of cardiac arrhythmias secondary to hypoxia. The heart of the obese patient frequently shows changes specifically represented by hypertrophy secondary to increased circulatory volume with diastolic dysfunction, increased left atrial pressure, and a higher risk of atrial fibrillation. In these conditions, the changes induced by the pneumoperitoneum and the absorbed CO_2_ during laparoscopy are more pronounced than in non-obese individuals, leading to higher BP and heart rate values [31].

The pneumoperitoneum is created using CO_2_. The gas peritoneal absorption is rapid, causing a fast increase in the partial pressure of carbon dioxide (PCO_2_) in the first 15 min, which is quantified by the increase in EtCO_2_. This phase is followed by a plateau period or a decrease absorption phase [32]. To reduce EtCO_2_, the ventilatory frequency and/or tidal volume can be reduced, but with the concomitant maintenance of a *p* peak value < 40 mmHg, to avoid hyperbaric induced lung injury and a reduced venous return. The oxygen saturation SpO_2_ does not change, as it depends on the PEEP and FiO_2_. 

The extraperitoneal administration of CO_2_ is associated with a more rapid rise in PCO_2_ and increased postoperative maintenance due to a higher CO_2_ concentration, which is achieved in the extraperitoneal space but also the vascularity of this region. However, the maximum PCO_2_ value does not differ between intra- versus extraperitoneal insufflation and postoperatively increased concentrations [25].

For patients with COPD, specific medication must be administered before the surgical procedure to prevent potential complications induced by bronchospasm in conjunction with the increased absorption of CO_2_ from the peritoneum. If clinical bronchospasm phenomena occur preoperatively or postoperatively, the therapy of choice is inhalation therapy [33].

A beneficial CO_2_-infused effect has also been described. Compared to open surgery, a reduction in cytokines (tumor necrosis factor-alpha, interleukin-6, and acute-phase reactant (C-reactive protein)) produced secondary to tissue damage was observed [34].

A consequence of reduced venous return with increased afterload is an imbalance between the myocardial oxygen supply and demand [25].

A decreased cardiac output is due to decreased venous return following pneumoperitoneum and reverse Trendelenburg position and is more common in hypovolemic patients or patients with pre-existing cardiac pathology [35].

To these, one should add the changes associated with CO2 absorption. Minor hypercapnia (PCO_2_ of 45–50 mm Hg) is not associated with hemodynamic changes, but moderate or severe hypercapnia can cause direct and indirect changes. By direct mechanism, respiratory acidosis induced by increased PCO_2_ leads to myocardial depression and vasodilation, and by indirect mechanism, via catecholamine release, to increased oxygen consumption [32].

Pneumoperitoneum-induced hemodynamic changes return to normal values in less than 10 min [36]. In patients with cardiovascular pathology, these changes may persist for more than 65 min postoperatively. Up to 20% of the patients may experience heart failure within the first 3 h postprocedure, secondary to decreased peripheral vascular resistance, increased heart rate, cardiac index, ejection fraction, and left ventricular mechanical work [37].

In the past, coronary ischemic pathology was a contraindication for laryngoscopy. A study on patients with coronary artery disease and ASA III–IV, in whom only laparoscopic cholecystectomy was performed, showed that this technique, was safe if a low peritoneal pressure of 10–12 mmHg was used and a careful monitoring of hemodynamic parameters was employed [38].

In rare cases, all these hemodynamic consequences can lead to decreased perfusion in the coronary arteries and acute coronary syndrome. Cases of acute myocardial infarction have been described postoperatively after laparoscopic cholecystectomy, but before current guidelines for cardiac pathology in non-cardiac diseases [39].

In our study, a strong correlation was observed between intraoperative blood pressure values and sex, indicating that women, especially those of childbearing age, have a lower blood pressure than men. This can be explained by the increased concentration of estrogen in women of childbearing age, which induces a decrease in peripheral vascular resistance and antagonizes the effects of catecholamines produced as a result of renal vascular compression during pneumoperitoneum [40].

We also noticed that intraoperative blood pressure is dependent on patient’s age, with blood pressure being lower as age increases. This could be explained by the fact that older individuals are often on antihypertensive medication that can lower intraoperative blood pressure values. Other studies have not highlighted this association.

In a study by Ramos L.P.J., no statistical correlation was demonstrated between age and hemodynamic variations during laparoscopic cholecystectomy. However, in their study, a small variation in diastolic blood pressure during laparoscopic cholecystectomy was noted. One explanation for this discrepancy between their findings and ours could be due to differences in study population. Their study focused solely on elderly patients, whereas our study considered both elderly patients (>60 years old) and especially patients < 60 years old [41].

The results obtained by us support the existence of a reverse correlation between intraoperative heart rate and age, with heart rate being lower as patient’s age increases. One explanation could be that elderly patients more often have beta-blockers as part of their chronic medication regimen, which significantly reduce the intraoperative heart rate.

Additionally, in our study, we found a positive correlation between postoperative blood pressure and age, with blood pressure being higher with advancing age. This can be explained by the fact that up to the age of 50, there may be an increase in peripheral vascular resistance, which accounts for both the high values of systolic and diastolic blood pressure. Then, a plateau of diastolic values follows, with a continued increase in systolic blood pressure due to the intensification of arterial stiffness over time. After the age of 60, as a result of these pathophysiological changes, a discrepancy between the increase in systolic and diastolic blood pressure is observed [42].

The results obtained by us suggest a positive correlation between heart rate and smoking status, indicating that non-smokers have a lower heart rate, at values considered within normal limits. Although the World Health Organization (WHO) found that the risk of postoperative complications, especially pulmonary, is higher in smokers, there are studies that have shown that smoking does not induce an increase in pulmonary or upper airway complications postoperatively [43,44].

Although there were significant correlations between age and blood pressure, or heart rate, our patients’ outcomes were without intraoperative or postoperative complications. In our study, laparoscopic cholecystectomy was a safe procedure regardless of the patient’s age. This is consistent with other studies in the literature. Rosario Vecchio et al. followed a total of 161 patients over the age of 65 who underwent laparoscopic surgery, demonstrating the safety of this technique even in older patient groups [45].

Naito S et al. monitored preoperative, intraoperative, and postoperative clinical parameters in patients over the age of 85 who underwent laparoscopic cholecystectomy, compared to a control group under the age of 85. They demonstrated that this type of surgical intervention is relatively safe even in very elderly patients, provided that careful monitoring is conducted, especially in those with associated comorbidities [46].

In summary, based on the results, our study, although it was carried out on a group of patients with continuous hospitalization, who were followed 24 h postoperatively, demonstrated, as other studies have shown, that this technique can be performed safely during 24-h hospitalization [47].

### 3.1. Limitations

This research has several limitations. Firstly, as it is a retrospective study, it may be susceptible to bias in patient selection. Secondly, our study was limited to a single center, thus the applicability of our findings may be restricted. Thirdly, the sample size we utilized was small, which could result in an overestimation of effects and biased samples. Finally, the patients were only followed-up with for 24 h postoperatively.

### 3.2. Future Directions

First of all, the authors of this article would like to continue this study by enrolling a larger number of patients with ASA 3 class to analyze their hemodynamic changes.

Then, future research may be directed toward laparoscopic cholecystectomy with awake patients. There was a first case report in the literature in 2023 of laparoscopic cholecystectomy with an awake patient, which was performed in Italy. The advantages of this procedure are mainly related to reducing post-anesthesia effects and a faster postoperative recovery. The data support the safe use of epidural and spinal anesthesia. Anesthesia advantages include no airway manipulation, better postoperative analgesia, faster recovery with less vomiting and nausea, the maintenance of spontaneous breathing during surgery, and dealing with cases of increased general anesthetic risk, such as severe lung disease [48].

Given that hypothyroidism can lead to difficulties with extubation and prolonged mechanical ventilation, resulting in additional acid–base balance disturbances in these patients, we believe that monitoring patients with hypothyroidism undergoing laparoscopic cholecystectomy could be of interest. This is especially important considering that some studies show an association between autoimmune thyroiditis and cholecystitis [49].

It would be important to monitor patients’ evolution in the days following the surgical intervention, as there are studies showing gut dysbiosis and the need for probiotic use to prevent secondary diarrhea syndrome [50,51,52].

One important element that should be evaluated in a future study is postoperative patient satisfaction, which can be indirectly influence by the post-treatment experience [53].

Lastly, further studies are needed for extended laparoscopic cholecystectomy in gallbladder cancer (GBC), as there are studies indicating that such a procedure may not be inferior to open surgery with regard to oncological safety, long-term outcomes, and technical feasibility in early stages of GBC [54].

## 4. Conclusions

Our study summarizes a direct intraoperative correlation between blood pressure and gender, with women having lower blood pressure. A positive correlation was also observed between intraoperative blood pressure and BMI. In addition, an inverse correlation was demonstrated between age and intraoperative blood pressure and between age and intraoperative heart rate. Both postoperatively and preoperatively, direct correlations were found between blood pressure and age.

Patients undergoing laparoscopic cholecystectomy experienced significant hemodynamic changes with pneumoperitoneum, but this type of surgical intervention was safe for patients regardless of their age.

This study confirms that laparoscopic cholecystectomy is a safe method regardless of age, sex, and BMI, with indication of same-day surgery.

## Figures and Tables

**Table 1:** Descriptive analysis.

Variables	Frequency (*n*)	Percent (%)
Gender		
Famale	293	85.7
Male	49	14.3
BMI, kg/m^2^		
<18.5—underweight	21	6.1
18.6–24.9—Normal	156	45.6
25–29.9—overweight	114	33.3
30–34.9—obesity grade 1	42	12.3
35–39.9—obesity grade 2	8	2.3
>40—obesity grade 3	1	0.3
Age		
<40 years old	56	16.4
40–59 years old	183	53.5
>60 years old	103	30.1
Residence		
Urban	228	66.7
Rural	114	33.3
Smokers	61	17.8
Nonsmokers	281	82.2
ASA		
ASA I	12	3.5
ASA II	271	79.2
ASA III	58	17
ASA IV	1	0.3
Operator scheme		
Elective surgery	251	73.4
Emergency	91	26.6
Diagnosis of hypertension	137	40

**Table 2:** Analytical analysis.

	Intraoperative		Postoperative		Preoperative	
	correlation	Pearson Coefficient	correlation	Pearson Coefficient	correlation	Pearson Coefficient
Blood pressure	direct correlation: intraoperative minimum blood pressure levels and sex (men having higher blood pressure)	(*p* 0.015 < 0.05)	direct correlation: postoperative maximum blood pressure levels and age	(*p* < 0.001 < 0.05)	direct correlation: maximum blood pressure value and BMI	(*p* < 0.001 < 0.05)
	direct correlation: intraoperative maximum blood pressure and body mass index (BMI)	(*p* 0.025 < 0.05)	direct correlation: postoperative maximum blood pressure levels and sex (male sex are associated with a higher maximum blood pressure value)	(*p* 0.009 < 0.05)	correlation between the preoperative maximum blood pressure value and smoking status	(*p* < 0.001 < 0.05)
	inverse correlation: intraoperative maximum blood pressure and age	(*p* 0.001 < 0.05)			direct correlation: minimum and maximum blood pressure and age	(*p* 0.001 < 0.05)
Heart rate	indirect correlation: maximum intraoperative heart rate and age	(*p* 0.015 < 0.05)	Correlation: minimum heart rate and smoking status (average value being higher in non-smokers)	(*p* 0.011 < 0.05)		

## Data Availability

The original data presented in the study are openly available on Figshare at https://doi.org/10.6084/m9.figshare.26046145.

## Abbreviations

The following abbreviations are used in this manuscript:

ASA	American Society of Anesthesiologists Physical Status
BMI	Body Mass Index
BP	Blood Pressure
CO_2_	Carbon Dioxide
COPD	Chronic Obstructive Pulmonary Disease
ETCO_2_	End Tidal CO_2_
FiO_2_	Oxygen Fraction
GBC	Gallbladder Cancer
HR	Heart Rate
PCO_2_	Partial Pressure of Carbon Dioxide
PEEP	Positive End Expiratory Pressure
SaO_2_	Oxygen Saturation
SPSS	Statistical Package for Social Sciences
